# The spatial coupling effect between urban street network’s centrality and collection & delivery points: A spatial design network analysis-based study

**DOI:** 10.1371/journal.pone.0251093

**Published:** 2021-05-06

**Authors:** Muhammad Sajid Mehmood, Gang Li, Annan Jin, Adnanul Rehman, V. P. I. S. Wijeratne, Zeeshan Zafar, Ahsan Riaz Khan, Fahad Ali Khan

**Affiliations:** 1 College of Urban and Environmental Sciences, Northwest University, Xi’an, PR China; 2 Shaanxi Key Laboratory of Earth Surface System and Environmental Carrying Capacity, Northwest University, Xi’an, PR China; 3 Department of Geography, University of Colombo, Colombo, Sri Lanka; 4 Department of Environmental Sciences, Bahauddin Zakariya University, Multan, Pakistan; Institute for Advanced Sustainability Studies, GERMANY

## Abstract

The sustainable development of collection and delivery points and urban street network is an important consideration of logistic planners. Urban street networks have a significant impact on collection and delivery points’ location, but the spatial relationship between the centrality of urban street network and collection and delivery points has not been studied using spatial design network analysis. In a multiple centrality assessment model, we used point of interest and street network data to evaluate the location of two types of collection and delivery points and the centrality of streets in Nanjing city, based on four indicators: closeness, betweenness, severance, and efficiency. Then, kernel density estimation and spatial autocorrelation are used to study spatial patterns of distribution and centrality coupling effects of urban street network and collection and delivery points. The results show that the centrality of Nanjing streets has a big influence on the location of the collection and delivery points, and the directions of different types of centrality also vary. The location of the Cainiao Stations are largely related to closeness, followed by betweenness, severance, and efficiency. China Post Stations and street centrality have a weak correlation between efficiency and severance, but no correlation between closeness and betweenness. Our results can help logistics enterprises and urban planners to develop collection and delivery points’ network based on the urban street network.

## 1. Introduction

With the rapid development of information technology and e-commerce, the internet economy has become a new force in China’s economic development. Online trading is driving the rapid development of the offline logistics industry, which also makes the "last mile” distribution issue [1, 2]. In order to solve this problem, logistic enterprises created collection and delivery points (CDPs) to deliver goods. CDPs are third-party locations which are well-defined as the last stop of an enterprise where people can retrieve/return products, purchased over the Internet. Considered it the most cost-effective option which have become an alternate of parcel delivery system [3, 4]. As urbanization accelerates and online shopping trend increases, saving time is an important factor influence on parcel delivery. However, major cities such as Nanjing face serious traffic problems and delivery restriction of CDPs. Therefore, accessibility has become an important factor when choosing a CDPs’ location. There is no doubt that CDPs have become logistics of last-mile delivery in the city, which generate huge delivery volume and become even more big network of online shopping. CDPs are linked with streets, where logistic companies can install and accelerate the availability of CDPs’ networks [5–7].

The urban street network (USN) composed of roads, known as a skeleton of the city. With progress of time and network science, road networks have a clear geometric structure, composed of points (nodes) and lines (segments) [8, 9]. Transportation system mainly depends upon infrastructure of the roads which plays an important role for economic activities. Assessing the efficiency and convenience of any residential community relies on the road compatibility [10]. However, centrality is the basic concept of network science and graph theory, defining the most essential apexes of a graph, such as the main nodes of an urban road network infrastructure. Centrality mostly favours the location of the city and plays an important role in determining variations in the road networks for economic activities [11–13].

Road networks centrality has a direct spatial coupling effect with economic activities in urban cities [14]. Moreover, being centered CDPs are an image of development related to economic activities in the China. Thus, it reflects the significance of street networks centrality and its spatial relationship effect with CDPs. Therefore, centrality is vital bridge to study the effects of spatial connections between urban space and CDPs. Objective of this research is to explore the correlation effect between centrality and CPDs.

A network data base developed by ArcGIS software with a help of vector road line and CPDs location. After that centrality was measured using multiple centrality assessment (MCA) model through spatial design network analysis (sDNA) tool. On the basis of centrality elements (closeness, betweenness, severance and efficacy), spatial interpolation was done by using Kernel Density Estimation (KDE) method. Further KDE of CDPs was analysed and transform on to the same scale map. Band collection statistics were also performed for CDPs and street network centrality with respect to Nanjing City, China as a research study.

The results include recommendations for planning and building urban street networks, as well as optimization and scientific positioning of the CDPs to consider network centrality. Social economy and CDPs facilitated by coordination growth.

**Fig 1:**
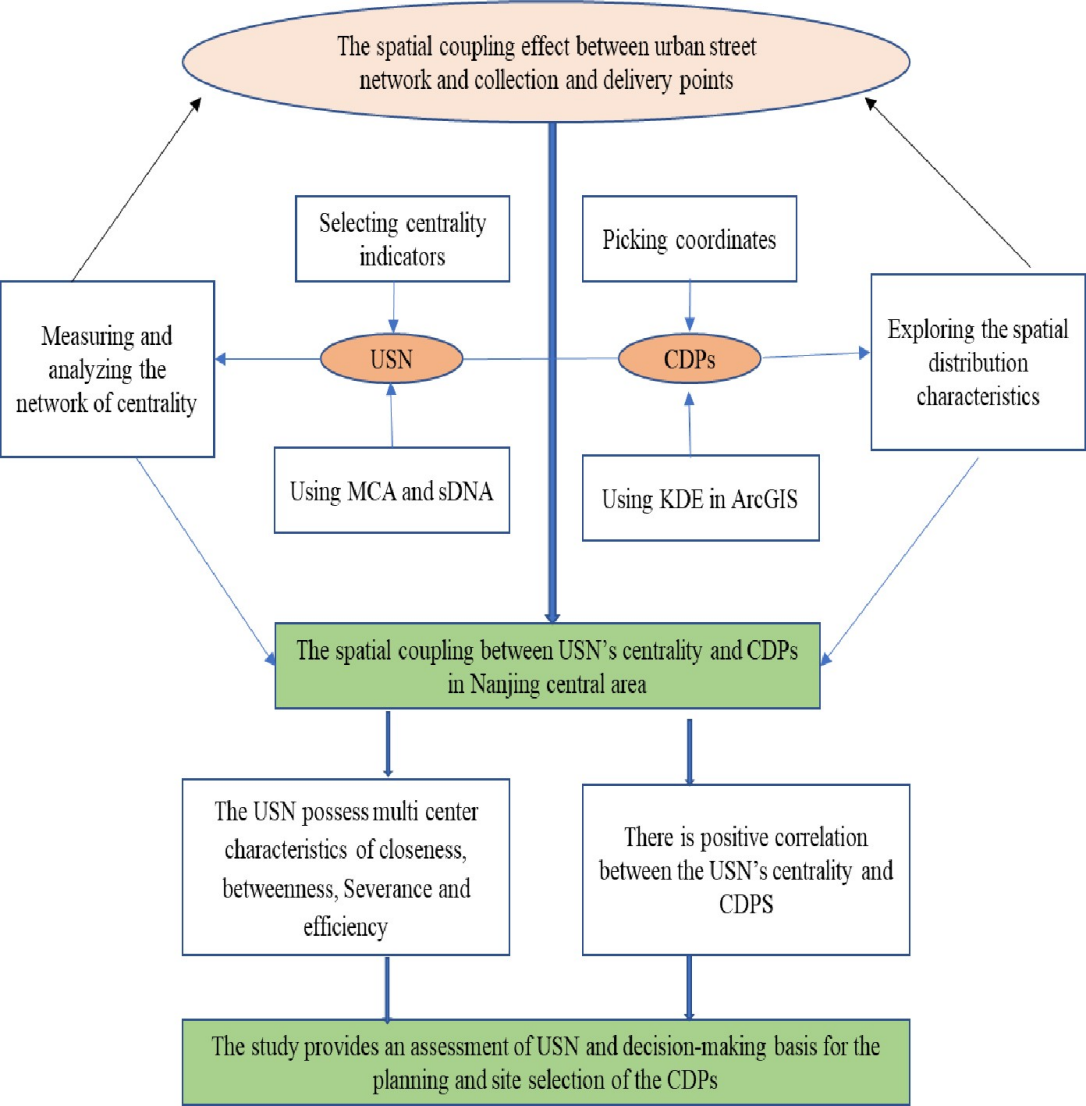
Research framework.

Remaining article has following sections in which 2nd part presents research techniques such as MCA, KDE, and spatial autocorrelation techniques. In 3rd part elaboration on the spatial distribution characteristics and spatial coupling effects of USN and CDPs. Final part, presents the policy recommendations made for policymakers and conclusions of the study. The research framework of this study is partially modified from Ma et al (2019) which is shown in Fig 1 [15].

## 2. Literature review

### 2.1. Association between the street network and various activities

Various activities like commercial stores, public health facilities, urban land use and recreational activities are associated with street network which are based on recent studies in the field of geography by many researchers to explore the relationship among each other. For example. He et al (2019) explored street networks and recreational activities’ locations. They found that closeness and betweenness cannot be used for a good road network design, but severance and efficiency are also important. The four street network indicators are spatially related to recreational and leisure activities. Different types of leisure and entertainment activities have different preferences for location on the street [16]. Ahmadzai et al (2019) studied road network evaluation and modelling based on the Integrated Graph of Natural Road Network. The results show that the weight of the edge is very favourable to the centralities of the betweenness and straightness. Weights that represent connections between nodes and specific locations are more important for accuracy near the closeness centre [17].

Some studies have explored the association between economic activities & urban land use and the different street centrality indexes. Porta et al (2009) used multiple centrality assessment models (MCA) to capture road centrality indices at various locations in Bologna, Italy and found that the retail stores’ location was highly linked to centrality [18, 19]. Porta et al (2012b) analysed the economic activities’ locations and street networks and expressed a strong relationship between street centres and various types of economic activities, and the results showed that the relationship between secondary activities and street centres is closer than the main types of activities [10]. Rui & Ban (2014) explored the association between street centrality and land use. The results show that there is a significant positive correlation between street centrality and urban land use [20].

Zhang et al (2019) explored the commercial stores’ locations in Beijing from the perspective of centralities of the street. They found that in three store types (all shops, restaurants, cafes, and bars) with correlations of 0.5, the correlation of all weighted centrality indices except straightness was high and the weights of landmarks’ correlation coefficient were the highest. The most important centrality indicator in this study was the closeness that was followed by betweenness. [21]. Han et al (2019) identified in the study of road network structure, retail stores’ spatial patterns are mostly correlated with closeness [22].

Yin et al (2018) analysed the centrality of roads and urban landscapes in Wuhan. As a result, it was shown that there is a strong connection between the street centrality and the urban landscape existed (the higher the centrality, the more fragmented the urban landscape) [23]. Wang et al (2018) evaluated point of interest (POI) data & street centrality and land use density in Shenzhen. They concluded that in terms of measuring locational advantages, closeness and straightness are more capable than betweenness. On a larger scale, centrality and intensity are more positively correlated [12, 24].

Lin et al (2018) conducted a study in Guangzhou, China between the retail stores’ locations and centralities of the street. They believed that all outlets are closely connected with high closeness, while retail stores were distributed in better betweenness areas [25]. Ma et al (2019) studied the network centrality (i.e., closeness, betweenness, straightness) regarding commercial complexes and the spatial coupling effect of urban public transport. The results showed that UPTN has multiple centralities according to transport routes. Moreover, in the city business areas, urban commercial complexes are mainly distributed, which showed a linear positive correlation with UPTN’s centrality [15].

### 2.2. Street network and its relationship with CPDs

There is an extensive literature on investigating the connection between CDPs’ location and conveyance. Researchers have shown that transportation formations such as the road structure and community transport can primarily affect the CDPs’ distribution. Moreover, street networks shape the connectivity, intensity, and distribution of CDPs. Also, street network is a key factor affecting the placement of CDPs [26–33].

Frequent resources storages, energy efficiency, environmental crisis and complex functionality of space are proposed to develop modern CDPs’ network integration. Important factors like traffic flow lines, regional distribution and CDPs’ layout should be focused on street network [34–37]. Modification and optimization of road network in street planning may helpful for ensuring the accessibility and connectivity of CDPs’ layout [35, 38]. Contrary to this road network spatial layout also affect the delivery flow and CDPs’ location. For example, CDP’ located on the primary roads, has easy to access the delivery volume and convenient for consumers to collect their parcels [39, 40].

Thus, existing research shows an organic relationship between different activities and urban street networks. These studies primarily examined the spatial distribution of specific activities using the urban network analysis (UNA) tool for the impact of interactions between activities and the street network’s centrality. According to literature, most of the above researches used the Urban Network Analysis (UNA) tool but not the Spatial Design Network Analysis (sDNA) tool. However, there are currently no studies on the relationship between sDNA-based street centrality indicators and CDPs, which is an effective way to measure street centrality. Thus, this study explores the coordinated and combined development of the USN and CDPs from a network centralization perspective.

## 3. Description of the study area and data sources

### 3.1. Description of the study area

**Fig 2:**
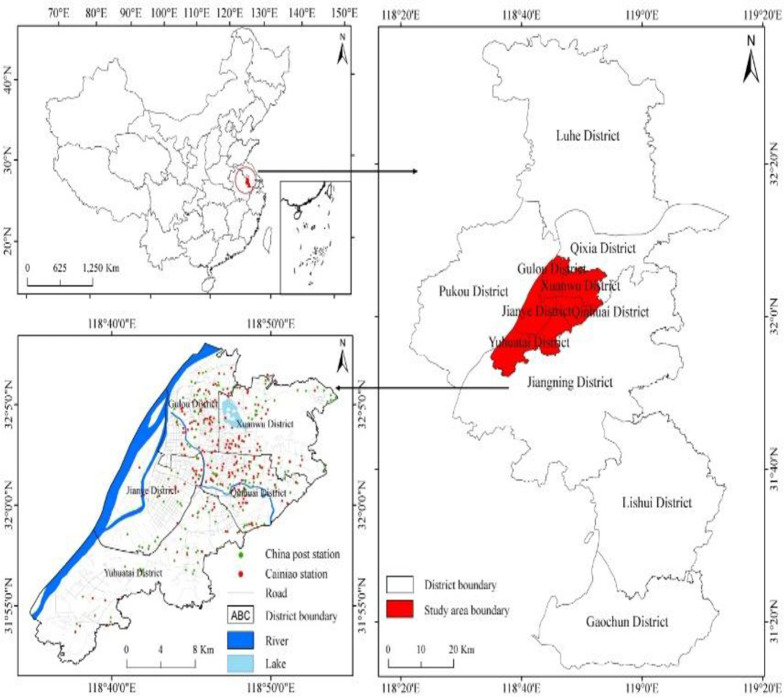
Study area.

Nanjing is located in the eastern Yangtze River Delta, China which is the second-largest city in this region and capital of Jiangsu province. It has 11 districts (**see Fig 2**) with a total area of 6,587 km^2^. The city has a population of 8.5 million and the urbanization rate is 83.2%. Nanjing has been one of the largest cities in China for thousands of years and is known as one of China’s capital among four ancient cities. Nanjing was the capital of the Chinese dynasties from the 3rd century to 1949, a major centre of culture, research, education, economy, politics, tourism, transport, and having one of the largest inland ports in the world. Nanjing is famous for its cultural and historical landscapes, including Confucius Temple, Drum Tower, Chaotian Palace, Porcelain Tower, Ming Palace, Stone City, Xuanwu Lake, City Wall, Qinhuai River, Purple Mountain, etc. The main cultural sites include Nanjing Museum, Nanjing Library, and Nanjing Art Museum. Our research focuses on the centre of Nanjing, which covers an area of 400.57 square kilometres, where 47.4% of the population resides. Over 43.66% of CDPs are concentrated in urban areas of Nanjing.

### 3.2. Data sources

Using POIs query to extract CDPs data from Metro Data Technology (MDT) (https://www.metrodata.cn/poi) and create a database by extracting street network data from Baidu and Gaode Map (www.baidu.com, https://www.amap.com/). The final data set includes 534 CDPs in research, including 237 Cainiao Stations and 297 China Post Stations.

- China Post is a state-owned enterprise operating the official postal service of China, providing postal services in mainland China. The company officially shares the office with the State Post Office, a state agency at the branch level, which theoretically regulates the postal industry across the country.
- Cainiao Network is an enterprise founded by Alibaba Group on May 28, 2013 and based on Alibaba Group’s logistics vision to fulfil consumer orders worldwide within 72–24 hours a day in China.

## 4. Methodologies

### 4.1. Multiple centrality assessment

In the MCA model, four key indicators were chosen to assess the different characteristics of street centrality: closeness, betweenness, severance, and efficiency networks [23, 41]. These metrics are calculated by using the sDNA toolbox in ArcGIS. The sDNA approach is a complex methodology adapted from conventional spatial syntax (http://www.cardiff.ac.uk/sdna/) for the analysis of urban networks [42, 43].

1. Closeness represents the connectivity between the local part of space and all the other neighbourhood in the network and their accessibility. Paths near the destination are likely to be picked by individuals. Closeness is determined by the Euclidean average distance within the radius between the origin and all available destinations. The assumptions behind these measurements are the optimal building environment density of CDP facilities as well as the broad physiological barriers to creating distorted environments between the origin and its neighbourhood.
  $$C_{i}^{C}=\frac{N-1}{\sum_{j=1;j\neq i}^{N}d_{ij}}$$ (1)
  Where N is the number of total paths; i and j are nodes and links respectively. d is the Euclidean average distance between all paths.
2. Betweenness evaluates how each network link is filled by an entity as it moves through the network, thereby determining all potential routes through the network connection. By estimating the flow based on the shortest Euclidean distance, this coefficient is calculated and assumes that the CDP’s pedestrian flow reaches to its optimum level.
  $$C_{i}^{B}=\frac{1}{\left(N-1)(N-2\right)}\sum_{j=1;j\neq i}^{N}\frac{n_{jk(i)}}{n_{jk}}w_{j}$$ (2)
  Where N is the total numbers of paths in the networks; nkj is the number of shortest Euclidean distance between links k and j and njki is the number flows in the shortest paths between links k and j pass through path i.
3. Severance is the reverse relation of the network detour study. This aspect is reflected in the mean transfer rate and by calculating the distortion of the local network, which poses cognitive difficulty in navigation.
  $$C_{i}^{S}=\frac{1}{N-1}\sum_{j=1;j\neq i}^{N}\frac{d_{ij}^{Eucl}}{dij}w_{j}$$ (3)
  Where N is the number of total paths in the network. $d_{ij}^{Eucl}$ is the Euclidean distance between links I and j and dij is the mean transfer rate between links I and j.
4. Efficiency calculates links’ connectivity that covers distances in space or local areas. This coefficient can be measured by the convex hull maximum radius. This indicates that pedestrian interaction has many opportunities, and that pedestrian navigation is very effective.
  $$C_{i}^{E}=\sum_{j=1;j\neq i}^{N}n(i,j)w_{j}$$ (4)
  Where N is the total number of paths in network. N is the covered distance between links I and j; while w is the maximum radius measured with j links.

### 4.2. Kernel Density Estimation (KDE)

Kernel density estimation is a common nonparametric technique that uses spatial smoothing and interpolation to transform discrete event points or polylines into continuous surfaces. It is used to compute the discrete node density of output grid cells within different threshold ranges in the given area. The KDE calculation result has a smooth distribution, that reflects the law of distance reduction, and can more effectively produce changes in the nuclear density of the area. Density gradually decreases as the distance decreases; it decreases until the density at a distance becomes 0 [44]. Therefore, this study used the nuclear density estimation method to explore the gathering and dispersion of pickup points in space. The function is shown in Equation (3.1):

$$f(s)=\sum_{i}^{n}\frac{k}{r^{2}}\left(\frac{d_{is}}{r}\right)$$ (5)

Where *f(s)* is the density at position *S*; *r* is the radius of the search for the estimated core density; and *k* is the weight of point *i* to the position *S* distance *d*_*is*_. This paper uses methods for estimating core density to determine the allocation characteristics of on-site collection and distribution points on a city-wide basis.

### 4.3. Spatial autocorrelation analysis

Spatial autocorrelation describes the possible interdependence of variables distributed in a similar observation data zone [31, 32]. For aggregation, variance, or randomness in space, global spatial autocorrelation is used, defining the spatial characteristics or attributes of a specific phenomenon in the region. The most widely used spatial auto-correlation measures are Global Moran I, which demonstrates the distribution of regional attributes [33, 34].

In this process, we build a global model of spatial autocorrelation from two variables and explore the correlation between the CDPs and the street network’s centrality within the research area. The formula is given below.

$$I=\frac{\sum_{i=1}^{n}\sum_{j=1}^{n}C_{ij}(X_{i}^{a}-\bar{X_{a}})(X_{j}^{b}-\bar{X_{b}})}{\sum_{j=1}^{n}\sum_{j=1}^{n}C_{ij}\sum_{i=1}^{n}\left(X_{i}^{a}-\bar{X_{a}})(X_{j}^{b}-\bar{X_{b}}\right)}$$ (6)

Where I represent the Moran value index; *n* is the total number of spatial cells *C*_*ij*_ is a weight matrix that measures spatial cells and adjacencies between them; $X_{i}^{a}$ and $X_{j}^{b}$ are the attribute values where *a* is for spatial cell *i* and *b* shows the attribute value of spatial cell *j*. $\bar{X_{a}}$ and $\bar{X_{b}}$ are the average attribute value of *a* and *b*. The value of I is between -1 and 1. When value of I is greater than 0, it reflects positive spatial correlation. That is, high value is adjacent to high value and low value is adjacent to low value. When I is less than 0 then it shows negative spatial correlation. It means low value is adjacent to high value while high value is adjacent to low value. When I is equal to 0 then there is no spatial correlation and spatial distribution is random.

### 4.4. Pearson correlation

Correlation coefficient can accurately indicate the close relationship between the two variables, in order to explore the relationship between centrality indicators and CDPs using SPSS 22.0 software. Pearson’s correlation coefficient is the covariance of two variables divided by the product of their standard deviations, which displays the degree of linear correlation between variables. Pearson correlation coefficient also known as Pearson product-moment correlation coefficient which reflects the relationship between two variables. The formula is as follows:

$$r=\frac{\sum_{i=1}^{n}\left(x_{i}-\bar{x})(y_{i}-\bar{y}\right)}{\sqrt{\sum_{i=1}^{n}{\left(x_{i}-\bar{x}\right)}^{2}\sum_{i}^{n}{\left(y_{i}-\bar{y}\right)}^{2}}}$$ (7)

Where (x_i_, y_i_) are sample points of the two variables (x, y). The correlation coefficients are less than or equal to 1, where 1 is total positive linear correlation, 0 indicates no linear correlation, and -1 is total negative linear correlation. In this paper, Pearson correlation equals to 1 is used to determine the relationship between streets’ centralities and CDPs. where n is the sample size, which represents the observations and mean values of the two variables, with a range of values of 1, -1, and a value of 1

### 4.5. Regression analysis

A bivariate regression analysis was used to check the effect of street centralities on the CDPs. Model is represented as follow.

$$Y=\alpha_{i}+X_{i}\beta_{i}+\varepsilon_{i}$$ (8)

Y is dependent variable CDPs and *α*_*i*_ are constants while X_*i*_ are street centralities as independent variables, *β*_*i*_ are the coefficients and *ε*_*i*_ are the standard errors.

For China Post Stations

$$Y_{1}=\alpha_{i}+X_{1}\beta_{1}+X_{2}\beta_{2}+X_{3}\beta_{3}+X_{4}\beta_{4}+\varepsilon_{i}$$ (9)

For Cainiao Stations

$$Y_{2}=\alpha_{i}+X_{1}\beta_{1}+X_{2}\beta_{2}+X_{3}\beta_{3}+X_{4}\beta_{4}+\varepsilon_{i}$$ (10)

## 5. Research results and discussion

### 5.1. Spatial pattern of street network’s centralities

#### 5.1.1. Overall spatial distribution features

**Fig 3:**
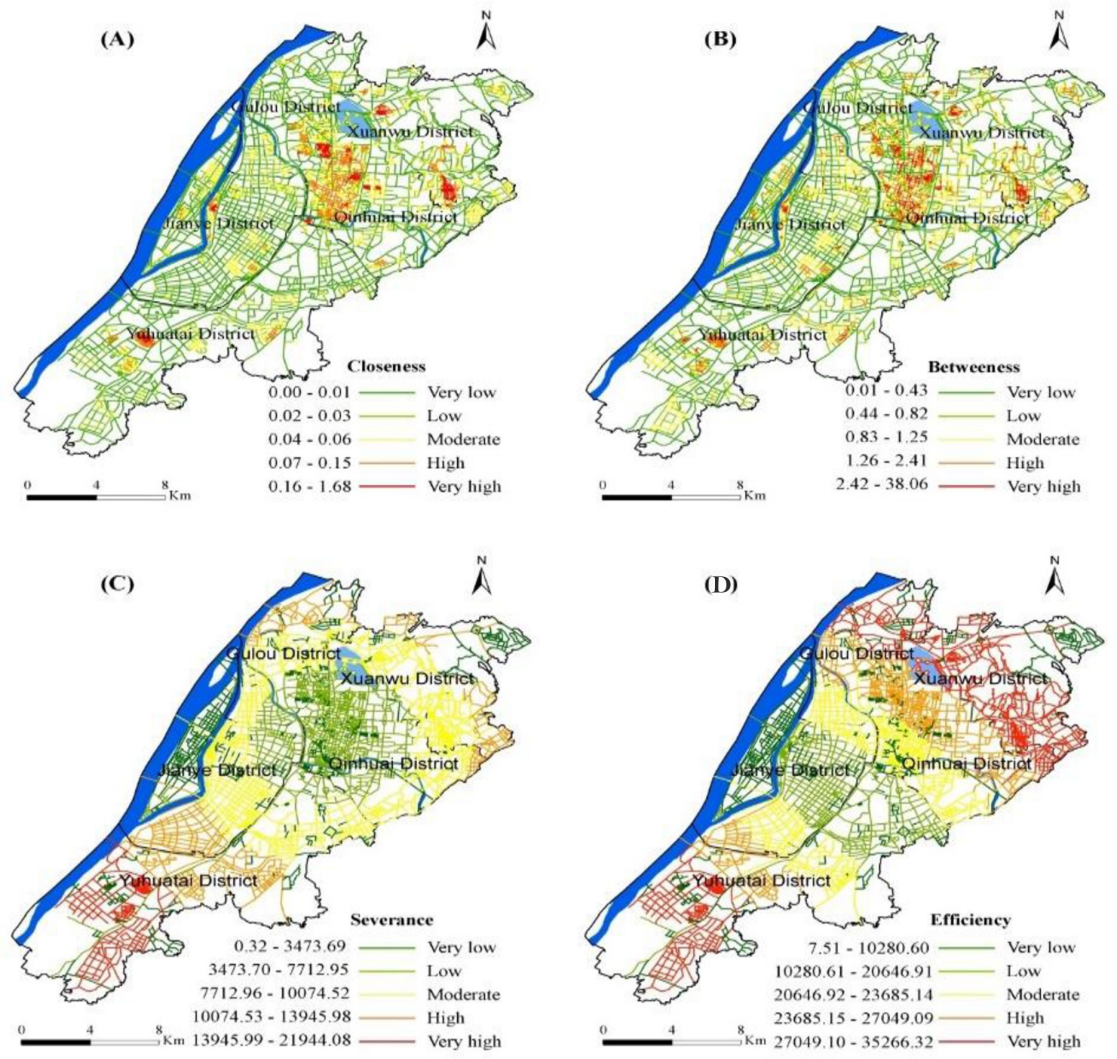
Spatial distribution of USN’s centralities in downtown Nanjing city.

When people move, they may have different perceptions of the configuration of the street network. To compare the influence of the street network index on the CDP location, four street network centralities were obtained from the sDNA software, namely closeness, betweenness, severance, and efficiency, based on the MCA theory. Fig 3 shows the spatial distribution characteristics of the four indicators in downtown Nanjing. Less distribution of severance of street networks will improve the viability of the city, creating more opportunities for people to move around. As shown in Fig 3, the street network in the urban fringe has less severance, and the neighbouring environment is suitable for the road network reduction. While an increase in the severance of the street network can interfere with connecting movement of city centres and suburbs.

The centrality of the streets in the study area is characterized by a heterogeneous spatial pattern, so with a higher central concentration and a multipolar distribution in the suburbs indicates a decrease in density from the central area to the suburbs. The higher closeness of the location, average distance is shorter to the centre showing easy access. The highest global closeness values of nodes are often scattered in the geometric centre of the study area [35, 36], and determined the urban core area structure. In general, the closeness of the study area gradually decreases from the centre to the periphery. Highest values of closeness are placed near Xuanwu Lake Street in Xuanwu District, Ninghailu Street in Gulou District, Fuzimiao Street in Qinhuai District, Xinglong Street in Jianye District, and Xishanqiao Street in the middle section of Yuhuatai District. All these streets formed a central business area around the research area (Fig 3A). Results shows that the business regions show highest degree of closeness in the study area while these locations are nearest to all other sites in the network.

Betweenness represents the possibility of an intermediary place to attract traffic. Higher the nature of the betweenness indicates paths are shorten through it, and more prominent will be its value. The distribution of betweenness in the study area is concentric, with the largest value near the city centre and decreases when moving outward which resembles closeness. The highest betweenness values are located in the northwest of Xuanwu District, including Hongshan Street and southeast of Zhonghuamen Street in Qinhuai District. These streets are connected to expressways (Fig 3B), indicating that Nanjing City Expressway plays a significant intermediary role in linking the shortest path.

The highest severance reflects a more direct link with all other locations and thus, allows higher transport efficiency than a lower severance. The highest severance nodes in the study area are relatively scattered across the southern and south-western of the Yuhuatai District regions. However, the regions with the severance are present in the southwestern part of the city centre, which demonstrates the “five industrial clusters of the city” (Fig 3C). The results show that new industrial zones play an important role in the road network.

The highest efficiency reflects more efficient navigation of connecting places with links and covering the distance. As shown in Fig 3D, the most effective links in the study area are relatively scattered in the north-western part of the Gulou District, the central part of Xuanwu, the north of the Qinhuai District, and a small part of the central and southern areas of Yuhuatai, connected to the suburbs of the research area.

Overall, the downtown of Nanjing has high street network centralities. However, under the influence of a rapid street network, the betweenness and severance show a trend of multi-polarity while suburban administrative centre has become the second-highest value areas.

#### 5.1.2. The spatial distribution of KDEs of street network’s centralities

**Fig 4:**
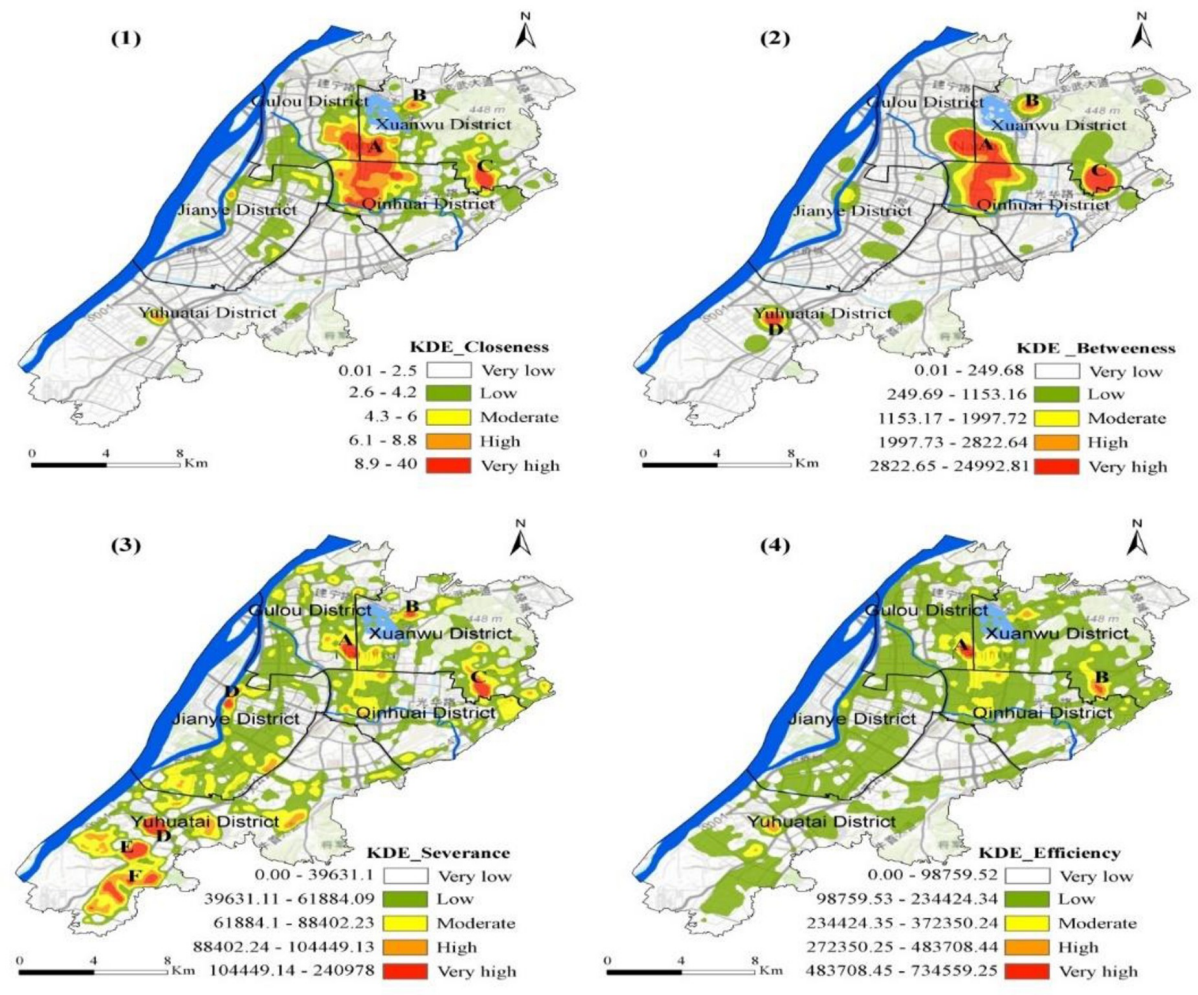
Spatial KDE distribution of USN’s centralities in downtown Nanjing city.

KDE of the street network’s centralities (closeness, betweenness, severance, and efficiency) were calculated according to their weightage. Fig 4 demonstrates the core density of the street network centrality in downtown Nanjing.

Fig 4(1) illustrates the distribution of the closeness kernel density in downtown Nanjing which displays central edge distribution pattern. The red area is the centre of the region with the highest kernel density representing regions A, B, and C. The central kernel density area is extended and diffused outwards, creating a lower kernel density area. The kernel density of closeness is between 8.9 and 40, including three regions which can be seen in graphical analysis. Where A is the intersection of the Gulou, Xuanwu, and Qinhuai areas. This area is the core centre of Nanjing and includes densely populated and public transport dense areas. In these areas, the density of roads is relatively high. Area B is a small space located in the Xuanwu area, west of the city. Area C is situated at the junction of the Qinhuai District and Xuanwu District, east of the Xuanwu District.

Fig 4(2) shows that the kernel density distribution of betweenness between street networks shows a centre-edge distribution very similar to the closeness density distribution. Four areas A, B, C, and D have the highest values of kernel density between 2822.65 and 24992.81. Zone A contains the intersections of the Gulou, Xuanwu, and Qinhuai districts. The kernel density for zone B is also high which extends from the Nanjing Railway, including Xuanwu Lake. Area C is in the east of the Xuanwu district and crosses the northern part of the Qinhuai district with smaller population and fewer roads. Region D belongs to the central area of the Yuhuatai district. The area contains Yuhuatai Martyrs Mausoleum which has a very less road distribution. In general, most of the passages pass through zone A and zone B. This shows that roads in this area occupy important positions in the entire road network. The distribution of roads in areas C and D is not enough, which indicates that roads in these areas are less important in the road network.

In general, the number of routes through area A and area B are the largest, implying that the streets in that area hold an important position in the entire street network. Roads distribution in areas C and D are relatively poor, suggesting that the streets in these areas have a lower road network status.

Fig 4(3) describes the severance core density distribution of Nanjing’s downtown street network with multi-centre characteristics. In regions A, B, C, D, E, F, and G, severance has the highest nuclear density, ranging from 104,449.14 to 240978. There are also various slight high-density areas around these regions. First, area A is situated in the east of the Gulou District, near the boundary of the Xuanwu District. Region B is located in the west of the Xuanwu District. Area C is the eastern part of the Xuanwu District. The kernel density value of the severance in these areas is the highest level. Areas with high kernel densities include the southern part of Jianye, which represents region D. Regions E and F are located in the middle of the Yuhuatai district. The G zone is in the east of Yuhuatai District. The street network in all these zones has a high degree of severance.

Fig 4(4) expresses that the distribution of kernel density efficiency in the street network has a central feature in the downtown of Nanjing. Two regions, A and B, have the highest values of kernel density in the range from 483708.45 to 734559.25. Zone A includes the eastern part of the Gulou district, which connects borders with Xuanwu District. Zone B is the eastern part of Xuanwu, not far from the border with the Qinhuai district.

### 5.2. Spatial patterns of CDPs

#### 5.2.1. Overall spatial distribution characteristics

**Fig 5:**
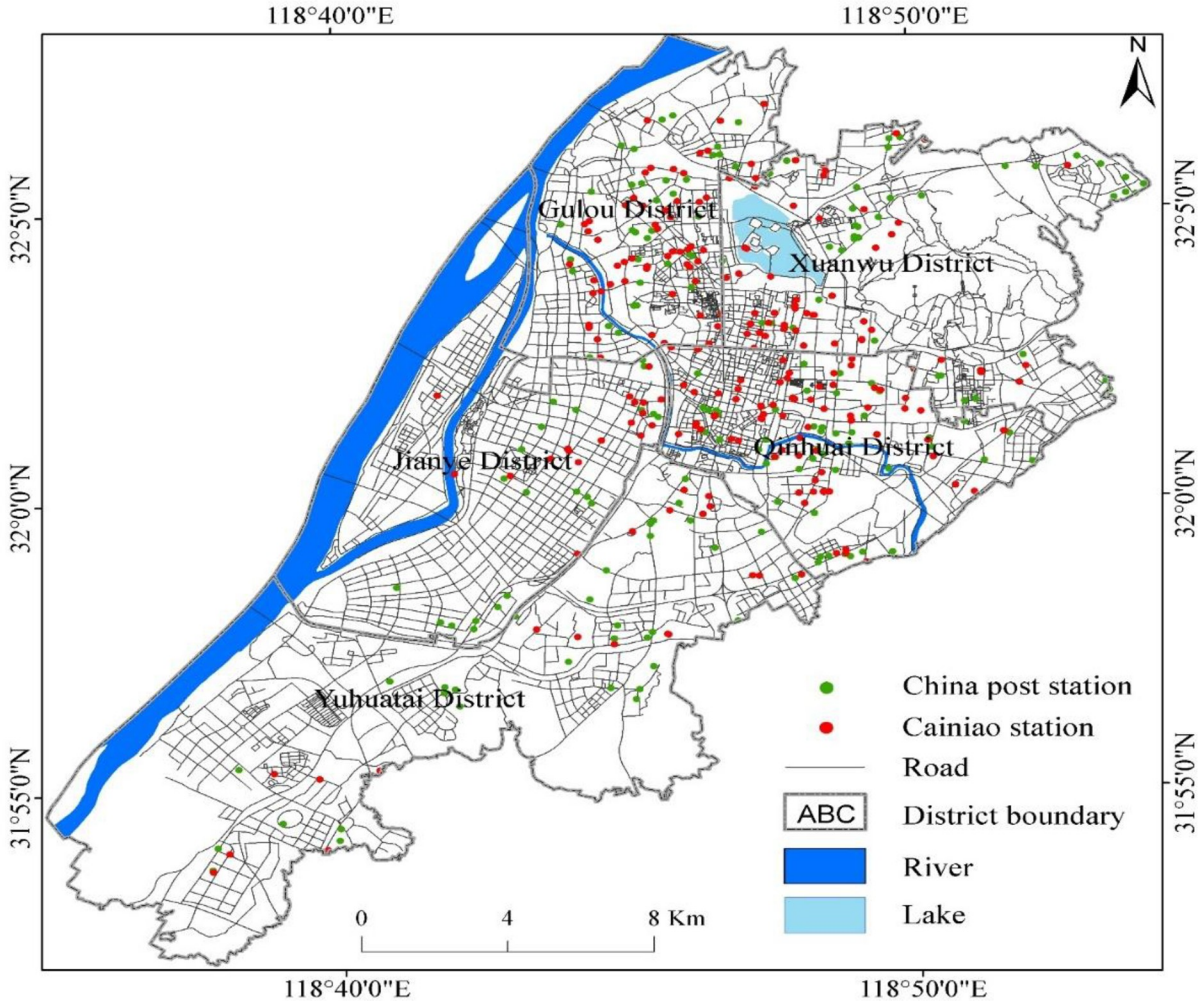
Spatial distribution of CDPs in downtown Nanjing city.

There are 534 CDPs in downtown Nanjing, including 237 Cainiao Stations and 297 China Post Stations. Using the Metro Tech website, we have built a data table containing the coordinates (latitude and longitude) of each CDP. Then data was imported into ArcGIS map to show the distribution of the CDPs in the downtown area of Nanjing (Fig 5). China Post Stations and Cainiao Stations are mainly distributed in Gulou District, Xuanwu District, and Qinhuai District, while Jianye District and Yuhuatai District have less distribution. The distribution pattern of China Post Stations is sparse, while Cainiao Stations are relatively concentrated.

#### 5.2.2. Spatial distribution characteristics of KDEs of CDPs

**Fig 6:**
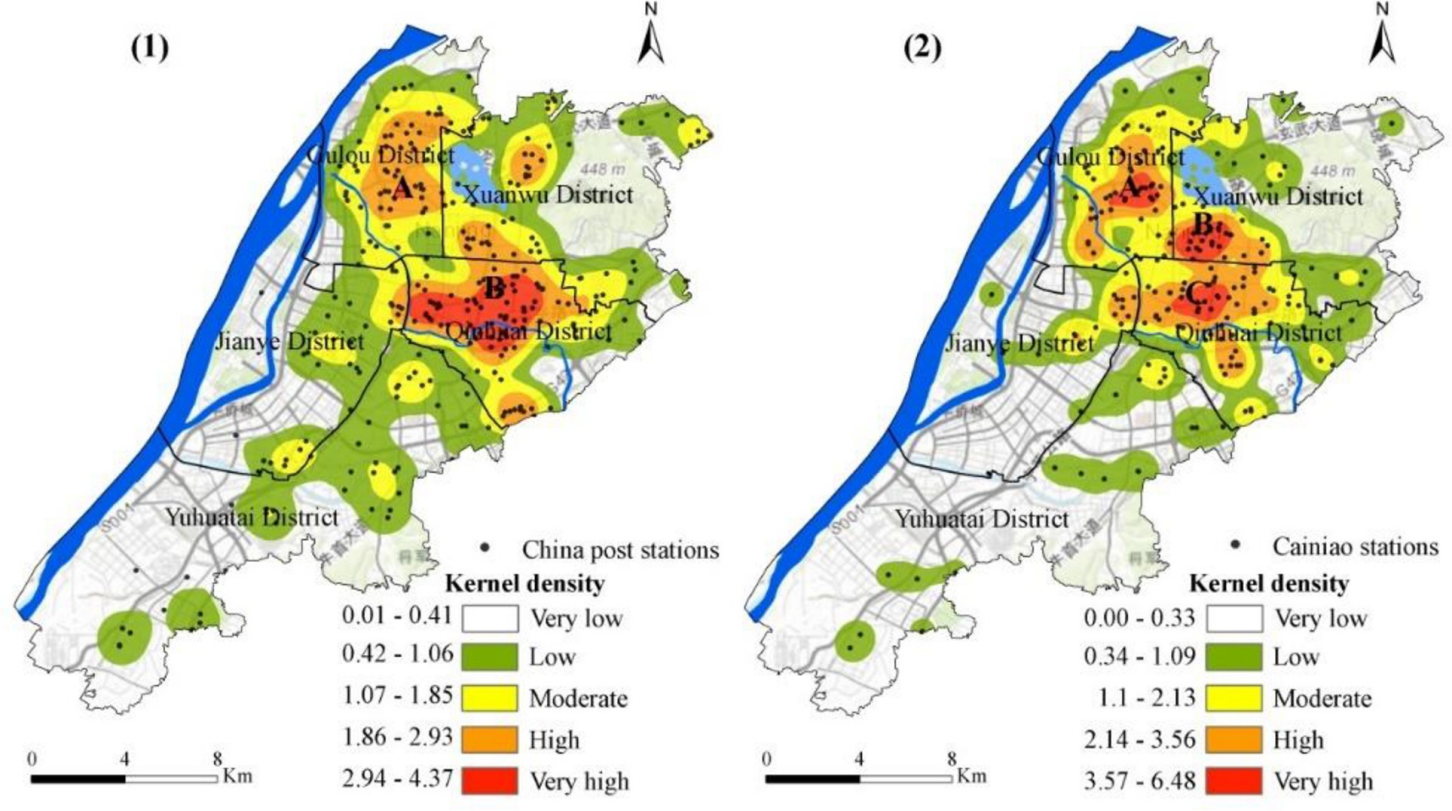
Spatial KDE distribution of CDPs in downtown Nanjing city.

Using ArcGIS’s KDE tool, the CDPs are uniformly mapped onto a grid with an equal spatial resolution of centrality index to cover the research area. In this way, CDPs’ kernel density in the centre of Nanjing is determined. It can be seen from Fig 6 that the distribution of CDPs is scattered.

In Fig 6(1), the distribution of China Post Stations is high in the centre of Gulou District and the Ninghai street, including residential streets and provincial government office areas as well as very high distribution in area B which is the central part of Qinhuai District.

The highest distribution of Cainiao Stations has three different regions like A, B, and C in different districts. Fig 6(2) shows that region A is located in the centre of the Gulou district, B is the south-eastern part of the Xuanwu district, while C is the centre of the Qinhuai district.

These two CDPs categories in central Nanjing are usually concentrated in areas with dense road networks. The distribution of CDPs in the Jianye and Yuhuatai areas is low and sparse. In terms of overall scale, the uneven distribution of CDPs is present in downtown Nanjing.

### 5.3. Spatial coupling effect analysis

CDPs’ spatial distribution kernel density characteristics and street network’s centrality in urban areas of Nanjing indicated that there is a spatial aggregation effect between them. Therefore, we further investigated the effect of the spatial relationship between the centrality of the street network and CDPs’ distribution.

#### 5.3.1. Spatial correlation analysis

The spatial correlation coefficient between the KDEs of street network’s centrality and the KDEs of CDPs was determined, using the Band Collection Statistic (BCS) method in ArcGIS software.

This section explores the relationship between KDEs of CDPs’ location and spatial disparities of KDEs of street’s centrality, and how the intensity of the association between the two CDPs’ categories and the KDEs of different centrality indicators are varied. Spatial correlation coefficients were calculated by obtaining the KDEs value of CDPs and the KDEs of centrality index of the gird cell in which the CDPs are located. Therefore, we performed a spatial correlation analysis of the KDEs of CDPs’ location and the KDEs of streets’ centralities.

Spatial correlation coefficients of KDEs of streets’ centralities (closeness, betweenness, severance, and efficiency) and KDEs of CDPs in Nanjing’s downtown area are shown in Table 1. There are four types of correlation approaches focused on band collection statistics: high correlation (0.8–1), significant correlation (0.5–0.8), low correlation (0.3–0.5) and weak correlation (0–0.3). The centrality of the street network has a weak, low, and significant spatial correlation with the KDEs distribution of Cainiao Stations. Specifically, the KDEs of closeness has the highest spatial correlation coefficient effect with KDEs of Cainiao Stations (0.573). In other words, Cainiao Stations are mostly distributed in these areas which have highest closeness value and the street network is relatively dense like Xinglong Street, Ninghailu Street, Xinjiekou Street, Xishanqiao Street, and Fuzimiao Street.

**Table 1:** Spatial correlation coefficients between street network centralities’ KDEs and CDPs.

KDEs of the CDPs	KDEs of the closeness	KDEs of the betweenness	KDEs of the severance	KDEs of the efficiency
**KDEs of the Cainiao Stations**	0.573	0.305	0.201	0.438
**KDEs of the China Post Stations**	0.490	0.238	0.206	0.407

All indicators are significant at level of 95%.

The spatial correlation coefficient between KDEs of betweenness and the Cainiao Stations is 0.305 which is low significant. This shows that the Cainiao Stations distribution is highly dependent on the proximity adjacent areas where density of streets’ betweenness is low. These streets are Xinglong Street, Ninghailu Street, Xinjiekou Street, Xishanqiao Street, and Fuzimiao Street. The average street network distance with stations is the shortest in these regions. On the other hand, areas such as Suojincun Street, Shazhou street, and Guxiong street have the lowest spatial correlation coefficient (0.201) between KDEs of the severance and Cainiao Stations. It means in these areas’ street network is scattered and the distribution of Cainiao Stations is also dispersed. The spatial correlation ratio of the KDEs of the closeness, efficiency and China Post Stations in the street network are 0.490 and 0.407, respectively which is low significant. Thus, the distribution of China Post Stations is affected by closeness and efficiency in that areas where density of street network is low such as Sai Hongqiao Street, Shuangzha Street, Fenghuang Street, and Zhonghuamen Street.

Moreover, the correlation between KDEs of the betweenness, severance and China Post is relatively small, respectively 0.238 and 0.206, Which shows that KDEs of betweenness and severance are present in some regions. However, these results are in a weak association and the distribution of China Post Stations is also low. In general, the different correlation coefficients reflect to some extent the influence of the USN’s central position on the spatial distribution of China Post Stations.

#### 5.3.2. Spatial autocorrelation analysis

**Fig 7:**
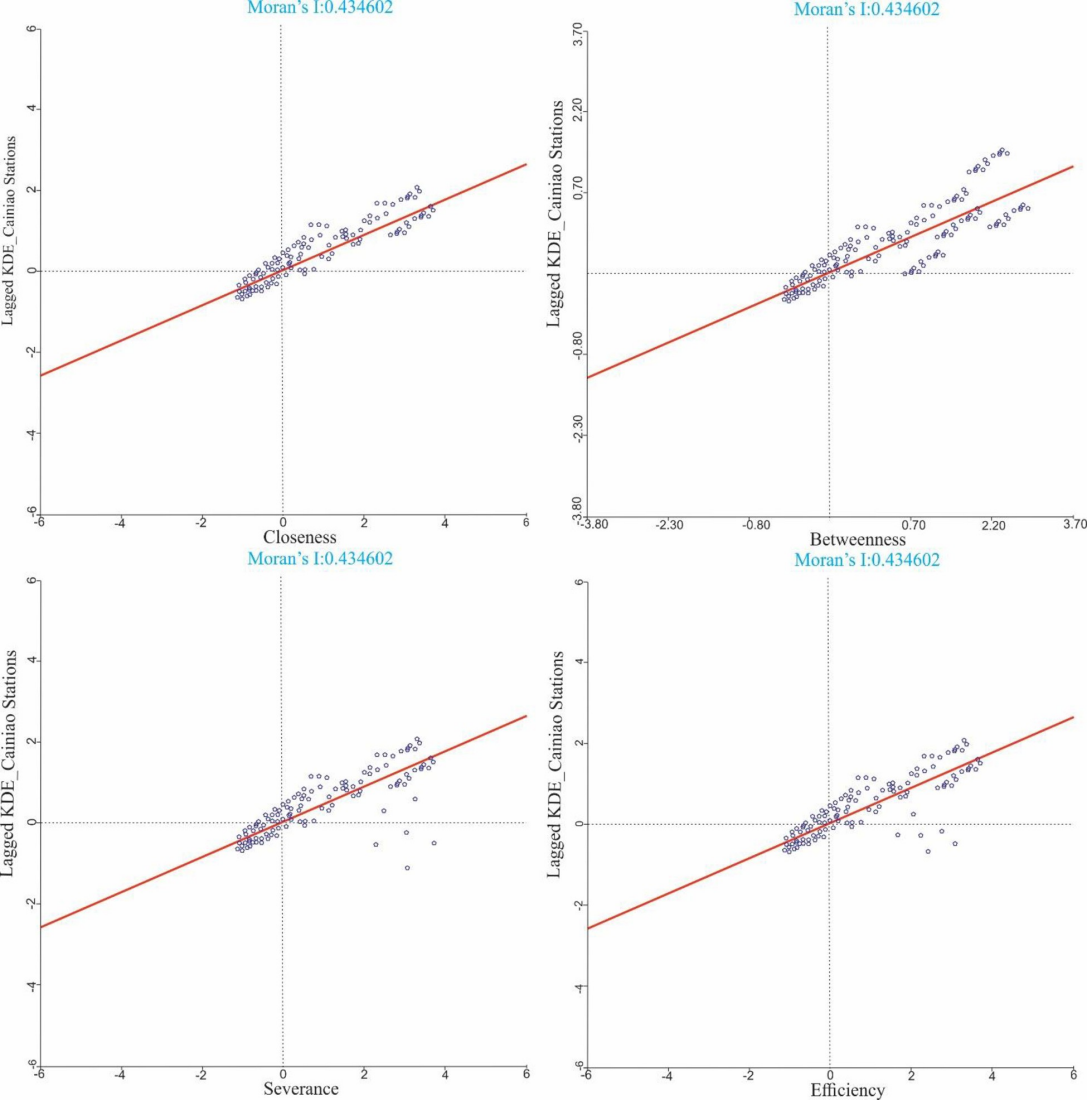
Moran scatter plot of the USN’s centralities and Cainiao Stations.

**Fig 8:**
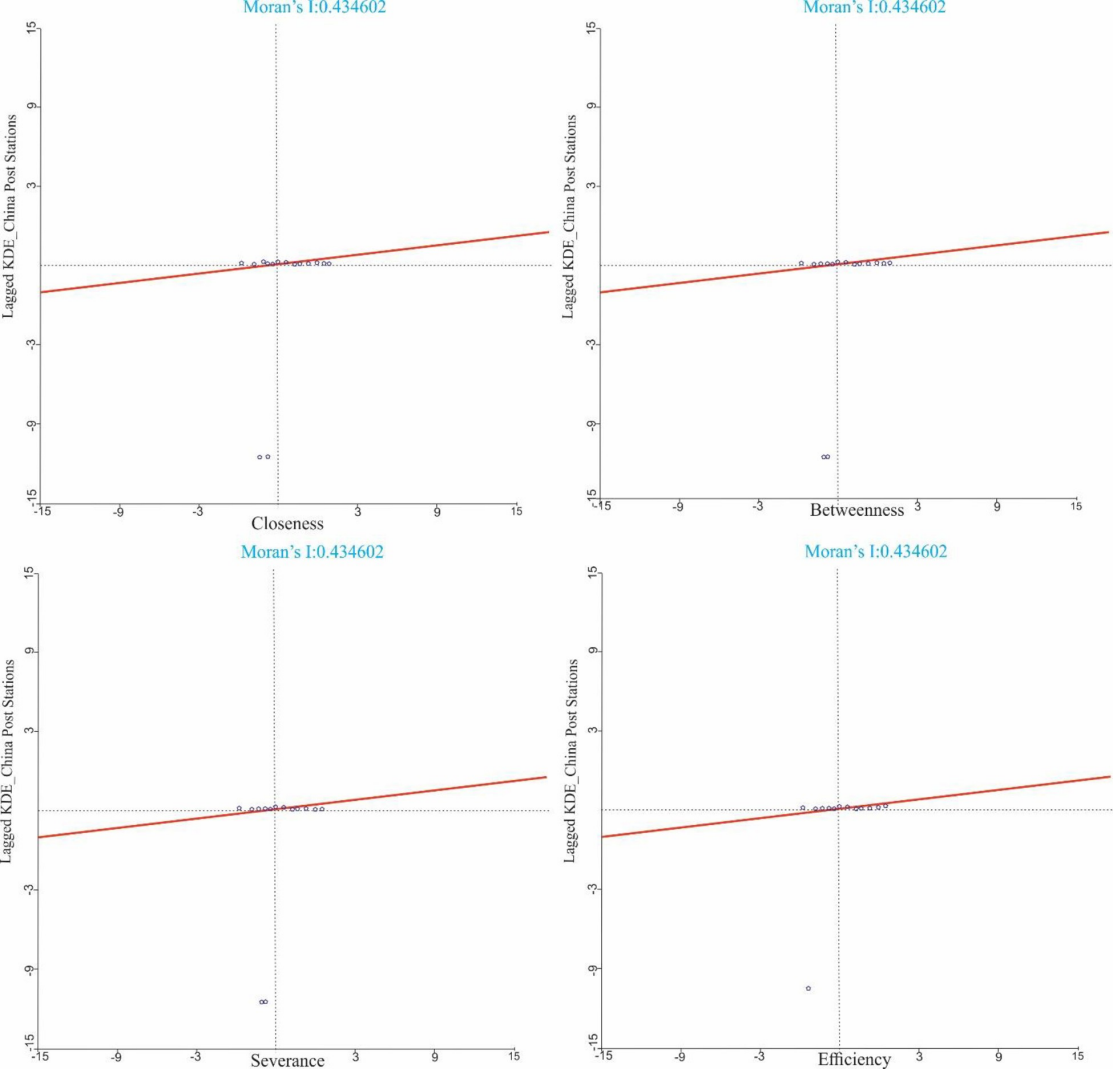
Moran scatter plot of the USN’s centralities and China Post Stations.

In this analysis, we used the spatial autocorrelation method to explain the spatial connection between street networks’ centrality and CDPs in the study area. First, ArcGIS was used to extract the kernel density value of the 534 CDPs and the kernel density value of USN’s centralities in the study area. After that, we used the Bivariate Moran I tool of the spatial analysis module to analyse spatial relationships using GeoDa software. The kernel density of the centrality of the street network was selected as the first variable and the CDPs’ values as the second variable. Then we created a Moran scatterplot that shows the relationship between the centralities of the road network and CDPs (Figs 7 and 8).

Fig 7 shows that the closeness, betweenness, severance, and efficiency of Moran’s I index of Cainiao Station are 0.434602, 0.435893, 0.245662, and 0.374621, respectively. All of these values are positive, indicating significant spatial correlation and clustering between the two indices. The p-value related to these findings is less than 0.01, which means that the spatial correlation and aggregation are very substantial. Based on these results, we concluded that the distribution of Cainiao Stations is clustered, because the street network is highly dense and accessible. These results are coherent with the analysis of spatial correlation findings. The KDEs of betweenness and Cainiao Stations have the largest Moran’s I index (see Fig 7(B)). The majority of nodes are clustered in quadrants 1 and 3 with high and low clustering. This also indicates that Cainiao Stations are mostly located in areas of high road accessibility. Moreover, the closeness KDEs Moran’s I index, and Cainiao Stations are also relatively large (see Fig 7(A)).

Cainiao Stations and KDEs of efficiency have a positive spatial correlation having Moran I index 0.374621 (see Fig 7(D)). The KDE efficiency is a possible load of each street on the street network. The higher the kernel density value, the greater the street network. This has contributed to an improvement in the flow of customers at Cainiao Stations. Specifically, Fig 7(C) indicates that the number of nodes in the first quadrant and the third quadrant is small. This is probably due to the low values of the kernel density in these areas. However, there were several Cainiao Stations in this area. As a result, the correlation between the severance and the Cainiao Stations is relatively low in terms of closeness, betweenness, and efficiency. It is concluded from analysis that the Cainiao Stations in the urban areas of Nanjing is often located in downtown with a good geographical location and an efficient network of urban roads.

Moran’s I index between centralities like closeness, betweenness, severance, and efficiency and China Post Stations are 0.068776, 0.066315, 0.181512, 0.148473, respectively are shown in Fig 8. The p-value is less than 0.001 associated with these results. All these values are positive, showing a weak spatial correlation and aggregation between the two indicators. Based on these findings, we concluded that the distribution of China Post Stations has a low density and inaccessible street network. These results are also consistent with the spatial correlation analysis’ results.

China Post Stations’ Moran’s I index value and severance KDEs are high (see Fig 8(B)). Most links are concentrated in quadrants 1 and 3 and are highly aggregated. It also shows that China Post Stations are often distributed in areas where a high road network is convenient. Also, the China Post Stations, and the Moran’s I index of efficiency KDEs are relatively large (see Fig 8(D)).

There is a positive weak correlation between the closeness and betweenness of KDEs and China Post Stations, with values of 0.066776 and 0.066315, respectively (see Fig 8(A) and 8(B)). These KDE values indicate that there are fewer links in the first and third quadrants. The agglomeration of China Post Stations is low. This also shows that China Post Stations are often distributed in areas with poor road accessibility. This is due to the sparse distribution of China Post Stations.

#### 5.3.3. Statistical correlation analysis

The above findings demonstrate a significant spatial association between the street network’s centrality and CDPs. However, we cannot determine the type of correlation (linear correlation or nonlinear correlation). Therefore, it is necessary to further analyse the statistical correlation. ArcGIS software was used to extract kernel density values for each CDP as well as for USN’s centralities. Then, further Pearson correlation and regression analysis were performed.

Pearson’s coefficients values between the KDEs of CDPs and KDEs of streets’ centralities are demonstrated in Table 2. and results are like spatial correlation analysis. The KDEs of severance and efficiency showed a significant linear correlation with CDPs. KDEs of Cainiao Stations had a significant linear correlation with KDEs of closeness, betweenness, and efficiency, while low correlation with KDE of severance, which are significant at a 95% level of confidence. Therefore, we can conclude that over all there is a significant linear correlation between the KDEs of street network’s centralities and KDEs of Cainiao Stations in downtown Nanjing.

**Table 2:** Pearson correlation analysis between KDEs of the centralities and KDEs of CDPs.

KDEs of CDPs	KDEs of Closeness	KDEs of Betweenness	KDEs of Severance	KDEs of Efficiency
**KDEs of China Post Stations**	0.075	0.066	0.182**	0.149**
**KDEs of Cainiao Stations**	0.600**	0.583**	0.312**	0.520**

**Correlation is significant at the 0.05 level.

Table 2 also shows that the correlation coefficients of KDEs of closeness and betweenness and KDEs of China Post Stations are non-significant, indicating that China Post Stations are distributed away from the shortest path where the KDEs value of closeness and betweenness are very low. These results are like spatial correlation findings.

Regression analysis was used to further study the relationships between these variables. We used the KDEs of CDPs’ value as a dependent variable (Y) and the street centralities’ KDEs value as an independent variable (X) including two types of CDPs and USN’s centralities; (a) China Post Stations and closeness, betweenness, severance, and efficiency, (b) Cainiao Stations and closeness, betweenness, severance, and efficiency to get the best fitting trend equation.

Regression results are consistent and passed the significance test for the KDEs of CDPs values and street network centrality. Again, these results prove that the location of the CDPs is highly dependent on the centrality of the roads. CDPs are often distributed across areas where the better centrality of the streets is present. However, except for Cainiao Stations and the street network, has best fitting connection between the China Post Stations’ distribution and the street centrality is not linear.

The relationship between the CDPs’ location and the centrality of the roads in downtown Nanjing may be determined by the locations of the CDPs as well as the street network. Table 3 displays the regression equations for CDPs and different centralities indicators in which the coefficient of determination (R^2^) is small and passed the significance test. The centrality indexes of the street network have been found greater influence on the distribution of CDPs which is an effect of a linear positive correlation. Among them, the spatial distribution of CDPs’ density is mainly influenced by the severance indicator. Also, the closeness correlation coefficient of the Cainiao Stations is very high, comparatively higher than the betweenness value. In other words, the closeness and betweenness indexes of the street network’s centrality have a significant effect on the spatial distribution of the Cainiao Stations. On the other hand, severance has low impact on the distribution of Cainiao Stations.

**Table 3:** Regression results between KDEs of centralities and CDPs.

Dependent variables (Y)	Independent variables (X)	p-values	Standard error	R^2^
**Cainiao Stations (Y**_**1**_**)**	Closeness (x_1_)	0.079*	1.357	0.470
	Betweenness (x_2_)	0.072*	1.506	0.360
	Severance (x_3_)	0.005*	1.534	0.195
	Efficiency (x_4_)	0.006*	1.304	0.380
**China Post Stations (Y**_**2**_**)**	Closeness (x_1_)	23.085	3.143	0.007
	Betweenness (x_2_)	5.547	2.751	0.003
	Severance (x_3_)	0.006*	1.585	0.123
	Efficiency (x_4_)	0.002*	1.710	0.126

* represents a significance level of 95%.

The closeness and betweenness correlation coefficient for China Post Stations are not fit in regression equations. The results show that closeness and betweenness have a significant impact on China Post Stations. This indicates that in the street network, there are fewer links to the China Post Stations for the shortest distance. Also, the correlation coefficients of severance and efficiency for China Post Stations are weakly fitted. This means that China Post Stations can be reached in these areas, but these areas have the capacity to develop new China Post Stations.

Analysis and results show that CDPs are mainly distributed along with the street network. The relationship between CDPs and indicators are more relevant because road efficiency affects residents’ willingness to travel and increases visits to CDPs. When designing and choosing locations, the average distance across all areas of the city-wide CDP network should be considered. Neighbouring kernel density can become more if a small average distance is required to achieve resident-CDP integration. Therefore, in high-density regions, the core density of the closeness to the CDP is high, and also the correlation coefficient between them is high.

## 6. Conclusions

The urban street network plays an indispensable role in city life. Centrality is a key feature in the formation and spatial allocation of socio-economic activities, and it plays a vital role in the growth of urban space. At the same time, USNs’ centrality has become an important impetus behind the transformation of sustainable urban CDPs’ network.

In the MCA model, two types of CDPs’ locations and street centralities (closeness, betweenness, severance, and efficiency) were used. Nanjing’s downtown area was analysed as an example using POI and street network data. This study draws the following conclusions.

The relationship of severance and efficiency with the China Post Stations is the weakest and does not correlate with closeness and betweenness. These types of CDPs are usually located in areas where long-distance routes are most commonly used. These areas can attract many consumers and satisfy the needs of many services for their purchase and delivery demands. They are mainly common in urban areas and populated suburbs.

The versatility of USN influences the accessibility of CDPs. The USN’s Centrality Index is positively correlated with the CDPs’ distribution. CDPs are mainly concentrated in urban areas with good geographical location and effective USN and differ greatly from region to region. For example, in this study, CDPs are mainly distributed in the areas of Ninghailu Street, Xinglong Street, Xishanqiao Street, Fuzimiao Street, and Xinjiekou Street. Therefore, the CDPs’ location can be chosen in areas with developed urban street networks, also the layout cannot be too concentrated to avoid traffic congestion. Thus, when choosing a location for a CDP and optimizing its location, city planners should comprehensively consider various influencing streets’ centralities that can effectively coordinate the construction of urban street networks and the development of CDPs.

We also know that the CDPs’ distribution is not only affected by the urban street network structure, but also by several factors, such as economic growth, industrial competitiveness, and the development priorities of CDPs in different cities. This study uses Nanjing, China as an example to verify the impact mechanism. The findings of this study apply to other cities of China, or at least those with similar city street networks.

The study made some valuable findings, but there are still some limitations. With limited data resources hinder the distribution of CDPs, including indicators such as CDPs’ revenue, operating area, workers capacity, the volumes of attended and unattended deliveries, and the effect of the urban public transport network. We mainly studied the spatial relationship between the USN’s centrality index and CDPs’ distribution. In the future, more CDP indicators may be included in the study to improve the generality of spatial linkage analysis.
